# Deep Brain Stimulation for Tremor Tractographic Versus Traditional (DISTINCT): Study Protocol of a Randomized Controlled Feasibility Trial

**DOI:** 10.2196/resprot.6885

**Published:** 2016-12-22

**Authors:** Bastian Elmar Alexander Sajonz, Florian Amtage, Peter Christoph Reinacher, Carolin Jenkner, Tobias Piroth, Jürgen Kätzler, Horst Urbach, Volker Arnd Coenen

**Affiliations:** ^1^Department of Stereotactic and Functional NeurosurgeryMedical Center, Faculty of MedicineUniversity of FreiburgFreiburgGermany; ^2^Department of NeurologyMedical Center, Faculty of MedicineUniversity of FreiburgFreiburgGermany; ^3^Clinical Trials Unit FreiburgMedical Center, Faculty of MedicineUniversity of FreiburgFreiburgGermany; ^4^Department of NeuroradiologyMedical Center, Faculty of MedicineUniversity of FreiburgFreiburgGermany

**Keywords:** deep brain stimulation, essential tremor, magnetic resonance tractographic-assisted implantation

## Abstract

**Background:**

Essential tremor is a movement disorder that can result in profound disability affecting the quality of life. Medically refractory essential tremor can be successfully reduced by deep brain stimulation (DBS) traditionally targeting the thalamic ventral intermediate nucleus (Vim). Although this structure can be identified with magnetic resonance (MR) imaging nowadays, Vim-DBS electrodes are still implanted in the awake patient with intraoperative tremor testing to achieve satisfactory tremor control. This can be attributed to the fact that the more effective target of DBS seems to be the stimulation of fiber tracts rather than subcortical nuclei like the Vim. There is evidence that current coverage of the dentatorubrothalamic tract (DRT) results in good tremor control in Vim-DBS. Diffusion tensor MR imaging (DTI) tractography-assisted stereotactic surgery targeting the DRT would therefore not rely on multiple trajectories and intraoperative tremor testing in the awake patient, bearing the potential of more patient comfort and reduced operation-related risks. This is the first randomized controlled trial comparing DTI tractography-assisted stereotactic surgery targeting the DRT in general anesthesia with stereotactic surgery of thalamic/subthalamic region as conventionally used.

**Objective:**

This clinical pilot trial aims at demonstrating safety of DTI tractography-assisted stereotactic surgery in general anesthesia and proving its equality compared to conventional stereotactic surgery with intraoperative testing in the awake patient.

**Methods:**

The Deep Brain Stimulation for Tremor Tractographic Versus Traditional (DISTINCT) trial is a single-center investigator-initiated, randomized, controlled, observer-blinded trial. A total of 24 patients with medically refractory essential tremor will be randomized to either DTI tractography-assisted stereotactic surgery targeting the DRT in general anesthesia or stereotactic surgery of the thalamic/subthalamic region as conventionally used. The primary objective is to assess the tremor reduction, obtained by the Fahn-Tolosa-Marin Tremor Rating Scale in the 2 treatment groups. Secondary objectives include (among others) assessing the quality of life, optimal electrode contact positions, and safety of the intervention. The study protocol has been approved by the independent ethics committee of the University of Freiburg.

**Results:**

Recruitment to the DISTINCT trial opened in September 2015 and is expected to close in June 2017. At the time of manuscript submission the trial is open to recruitment.

**Conclusions:**

The DISTINCT trial is the first to compare DTI tractography-assisted stereotactic surgery with target point of the DRT in general anesthesia to stereotactic surgery of the thalamic/subthalamic region as conventionally used. It can serve as a cornerstone for the evolving technique of DTI tractography-assisted stereotactic surgery.

**ClinicalTrial:**

ClinicalTrials.gov NCT02491554; https://clinicaltrials.gov/ct2/show/NCT02491554 (Archived by WebCite at http://www.webcitation.org/6mezLnB9D). German Clinical Trials Register DRKS00008913; http://drks-neu.uniklinik-freiburg.de/drks_web/navigate.do?navigationId=trial.HTML&TRIAL_ID=DRKS00008913 (Archived by WebCite at http://www.webcitation.org/6mezCtxhS).

## Introduction

Essential tremor is a hyperkinetic movement disorder characterized by a postural and kinetic tremor commonly affecting the upper extremities, but the voice, head, and lower extremities can also be involved [1]. Symptoms can result in profound disability and social withdrawal due to embarrassment affecting the quality of life [2]. In the stage of medically refractory essential tremor, deep brain stimulation (DBS) is an approved and safe procedure to achieve tremor reduction [3]. The historical target of DBS and stereotactic lesioning in essential tremor is the thalamic ventral intermediate nucleus (Vim) [4,5]. Pre–magnetic resonance imaging (MRI)–era localization of this structure was based on anterior commissure–posterior commissure (AC-PC) coordinates, and intraoperative DBS electrode placement relied on intraoperative electrophysiological analysis and tremor testing with multiple parallel trajectories in the awake patient to achieve satisfactory tremor control. Although the Vim is precisely identifiable with custom MRI nowadays, image-assisted DBS electrode placement in the Vim is still performed with a combination of the above mentioned indirect targeting procedures [6,7]. This can be attributed to the fact that the stimulation of fiber tracts seems to be more effective than stimulation of subcortical nuclei like the Vim [8], and indeed best results in tremor suppression are achieved with stimulation in the subthalamic region rather than in the Vim [9]. Recent diffusion tensor MR imaging (DTI) tractography studies revealed that the dentatorubrothalamic tract (DRT) is a promising target structure in the subthalamic region, as the current coverage of DRT fibers in Vim-DBS results in satisfactory tremor control [10-12]. DTI tractography-assisted stereotactic surgery targeting the DRT would therefore not rely on multiple parallel trajectories and intraoperative tremor testing in the awake patient. Moreover, DBS implantation in general anesthesia would offer more patient comfort and a shorter duration of surgery, reducing associated risks (eg, infection) [13]. The abandonment of multiple parallel trajectories and successive electrode manipulation during intraoperative testing should reduce the risk of intracerebral hemorrhage [14]. In a retrospective analysis, Chen et al [15] found that DBS for essential tremor with classical AC-PC coordinate-based targeting of the Vim can be performed in general anesthesia safely and effectively.

This clinical pilot trial, Deep Brain Stimulation for Tremor Tractographic Versus Traditional (DISTINCT), in patients with medically refractory essential tremor aims at demonstrating safety of DTI tractography-assisted stereotactic surgery in general anesthesia and proving its equality compared to conventional AC-PC coordinate-based stereotactic surgery with intraoperative testing in the awake patient. Accordingly, a randomized, controlled, rater-blinded, parallel group study was set up involving 2 patient groups each assigned to 1 of the aforementioned treatments. Established assessments of tremor and quality of life are used to compare the effect of treatments, which is then related to effective electrode contact positions.

## Methods

### Design

This is an investigator-initiated monocentric, randomized, controlled, 2-armed, interventional, observer-blinded feasibility trial conducted at the Department of Stereotactic and Functional Neurosurgery and the Department of Neurology at the Freiburg University Medical Center.

The primary objective is to compare DBS-mediated tremor reduction for 2 different approaches—DTI tractography-assisted stereotactic surgery with target point of the DRT in general anesthesia and stereotactic surgery of the thalamic/subthalamic region as conventionally used—in patients suffering from essential tremor.

Primary outcome parameter will be the reduction of tremor as assessed with the Fahn-Tolosa-Marin Tremor Rating Scale (FTMTRS) [16] including blinded video rating.

### Secondary Objectives

- To assess the effect in tremor reduction of both interventions at 6 months based on the total power in tremor analysis
- To assess the quality of life
- To assess the optimal electrode contact position
- To assess differences in duration of neurosurgery in both groups
- Psychiatric assessment
- To assess safety of intervention
- To show equality of both interventions in tremor reduction at 6 months based on the total power in tremor analysis

### Participants and Recruitment

We plan to recruit 24 male or female patients (12 per treatment group). Patients who are referred to our departments due to disabling medically resistant essential tremor are informed about this study. Patients who give their informed consent are registered in the trial and undergo the screening. Patients who gave their informed consent but do not undergo stereotactic surgery are regarded as screening failures. See Textbox 1 for eligibility criteria.

### Study Events and Assessments

**Figure 1:**
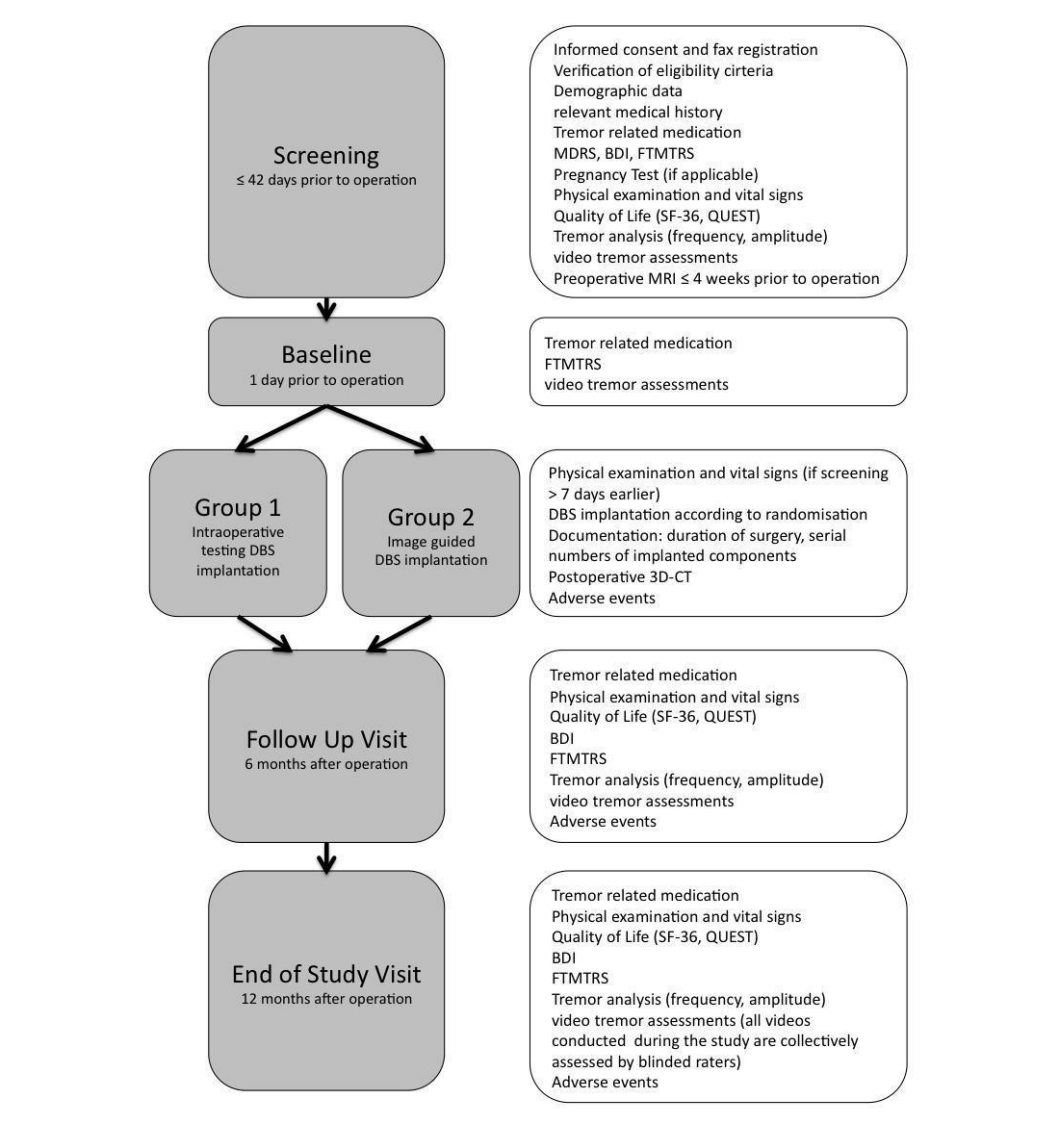
Schedule of study events and assessments. Study assessments on the right are conducted at the corresponding study events on the left. BDI: Beck Depression Inventory; CT: computed tomography; FTMTRS: Fahn-Tolosa-Marin Tremor Rating Scale; MDRS: Mattis Dementia Rating Scale; QUEST: Quality of Life in Essential Tremor Questionnaire.

A schedule of study events and assessments is provided in Figure 1.

**Eligibility criteria.**

Inclusion criteria:

- Aged 25 to 80 years, inclusive
- Essential tremor according to the criteria of the consensus statement of the Movement Disorder Society [17] with a medical treatment–resistant and disabling postural or intentional tremor
- Stable tremor medication for at least 3 months prior to inclusion
- An FTMTRS completed within 42 days prior to surgery
- Written informed consent

Exclusion criteria:

- Major depression
- Dementia (Mattis Dementia Rating Scale [MDRS]≤130)
- Acute psychosis
- Known or persistent abuse of medication, drugs, or alcohol
- Patient incapability
- Nursing care at home
- Surgical contraindications or medications that are likely to cause interactions in the opinion of the investigator
- Persons who are in a relationship of dependence/employment with the sponsor or the investigator
- Fertile women not using adequate contraceptive methods or with current or planned pregnancy or nursing period

#### Screening

Screening evaluations must be performed within 42 days prior to neurosurgery. For this evaluation, inclusion and exclusion criteria are checked and validated. The complete pretherapeutic work-up includes a physical examination, medical history, demography, vital signs, body weight and height, tremor-related medications list, quality-of-life questionnaires Short Form (SF)-36 and Quality of Life in Essential Tremor Questionnaire (QUEST) [18], MDRS, Beck Depression Inventory (BDI), FTMTRS [16], and tremor analysis (amplitude, frequency). A preoperative MRI is performed within 4 weeks prior to DBS implantation.

#### Baseline

Baseline visit will be performed on the day prior to neurosurgery and includes assessment of tremor-related medications, FTMTRS, and tremor analysis.

#### Tractography of the Dentatorubrothalamic Tract

As part of the preoperative diagnostic work-up, DTI is performed on a clinical 3 Tesla MRI system (Magnetom Trio, Siemens, Erlangen, Germany) with the following specifications: single-shot, 2-dimensional; spin-echo echo-planar imaging; repetition time (TR), 10,000 ms; echo time (TE), 94 ms; diffusion values, b=0 s/mm^2^and b=1000 s/mm^2^; diffusion directions, 61; slice count, 69; voxel size, 2.0×2.0×2.0 mm^3^; acquisition time, 11:40 min. Deformation correction of the EPI sequence is performed according to Zaitsev et al [19]. DTI tractography applying the fiber assignment by continuous tracking (FACT) algorithm is performed on a StealthViz workstation (Medtronic Navigation, Louisville, KY, USA) with the following parameters: fractional anisotropy level, 0.2; minimal fiber length, 10 mm; seed density, 5.0; maximal fiber cutoff angle, 50°. The seed region of interest (ROI) is placed in the ipsilateral dentate nucleus, while the target region is placed in the ipsilateral precentral gyrus.

#### Day of Neurosurgery

Patients will be randomized within 7 days prior to neurosurgery; the DBS system will be implanted according to the randomized treatment group.

#### Detailed Description of the Deep Brain Stimulation Implantation

After administration of standard antibiotic prophylaxis, a patient in a stereotactic frame (Leksell, Elekta, Stockholm, Sweden) is placed in local anesthesia in group 1 and under general anesthesia in group 2.

A computed tomography (CT) scan is performed, and the image data are transferred to the StealthViz Planning Station (Medtronic, Minneapolis, MN, USA). The previously acquired MRI sequences are coregistered to the stereotactic CT scan, and the trajectories are planned in group 1 (local anesthesia) based on AC-PC coordinates and imaging of thalamic and subthalamic region. The deepest point of the implantation will be the posterior subthalamic region or the caudal zona incerta, respectively, as visualized with a T2-weighted MRI sequence. With this technique a DBS electrode will traverse the Vim region (with the superficial contacts) while reaching the posterior subthalamic region with the most inferior contact. In group 2 (general anesthesia), based on imaging of the DRT, the subthalamic region will also be the inferior-most level of implantation.

In group 1, the bilateral DBS electrode implantation is performed under local anesthesia with the patient in a semisitting position. Using a microtargeting drive (MicroTargeting Star Drive M/E System, FHC Inc, Bowdoin, ME, USA), a test electrode (Cosman Medical, Inc, Burlington, MA, USA) is inserted through a frontal burr hole in the cranium. Macrostimulation is performed to confirm a contralateral clinical benefit (at a low threshold) and to test for side effects (at a high threshold). The definitive DBS electrodes are then implanted under fluoroscopic control. The implantable pulse generator (ACTIVA INS, Medtronic, Minneapolis, MN, USA) in group 1 is then implanted and connected to the DBS electrodes under general anesthesia.

In group 2, the bilateral DBS electrode implantation through a frontal burr hole via the planned trajectories is performed in ongoing general anesthesia under fluoroscopic control and without any further testing followed by the implantation of an implantable pulse generator (ACTIVA INS, Medtronic, Minneapolis, MN, USA), which is then connected to the DBS electrodes.

All patients in group 1 and 2 undergo a postoperative CT scan to corroborate the final DBS electrode localization. The following items will be documented at this visit: duration of surgery (time points of mounting frame, start surgery, stop surgery (= dismounting frame) and serial numbers of implanted components.

#### Follow-Up

At 6 and 12 months after neurosurgery, follow-up visits will be performed. The trial for the individual patient ends at the 12-month visit. On both visits, tremor-related medication, vital signs, quality of life (SF-36, QUEST), FTMTRS, tremor analysis (amplitude, frequency), and BDI will be assessed.

#### Video Assessments

The rating of the FTMTRS will be digitally recorded by video at every visit. The videotapes will be rated during the study period by an external group of movement disorder specialists who are not otherwise involved in the trial. To maintain blinding for the external raters, patients should wear headgear which completely encloses the hair.

### Biostatistical Planning and Analysis

#### Sample Size Calculation

This is a feasibility study in 24 patients with essential tremor (12 DTI tractography-assisted implantations, 12 implantations with conventional intraoperative testing). To obtain estimates for the treatment effect and its variance and, thus, to obtain a solid basis for the sample size calculation of the subsequent confirmative trial, the FTMTRS score at 6 months after intervention and its change from baseline will be subjected to exploratory descriptive analysis. Julious [20] found that a sample size of 12 per group in pilot studies seems reasonable for generation of pilot data. Given a continuous outcome, increasing the sample size beyond 12 per group did not have profound influence on the confidence interval. A dropout rate of 25% is assumed, so 30 patients will be assessed for eligibility.

#### Randomization

The randomization code will be generated by the clinical trials unit using the following procedure to ensure that treatment assignment is unbiased and concealed from patients and investigator staff. Randomization will be performed in blocks of variable length in a ratio of 1:1. The block lengths will be documented separately and will not be disclosed to the center.

#### Analysis of the Primary Endpoint

The primary efficacy analysis of this clinical trial will be conducted according to the intention-to-treat principle (ITT). This means that the patients will be analyzed in the treatment arms to which they were randomized, irrespective of whether they refused or discontinued the treatment or whether other protocol violations are revealed. Confidence intervals (95%) will be calculated for means and standard deviations within groups as well as for the difference between groups in the ITT population. The effects of DTI tractography-assisted versus conventional intraoperative testing with respect to the FTMTRS score at 6 months will be estimated and tested within a linear regression model including treatment and the baseline FTMTRS score as covariates. A conservative estimate of the effect size anticipated for a subsequent confirmative trial will be derived from these analyses by a combination of clinical and statistical judgement.

#### Analysis of Secondary Endpoints

Effective tremor reduction (FTMTRS score reduction by 50% is regarded as “response”) at 12 months after intervention will be analyzed in an exact logistic regression model. Tremor reduction measured by tremor analysis and calculation of total power before and 6 and 12 months after intervention will be analyzed the same way as the primary endpoint.

Quality of life will be measured with the SF-36. The 8 summary scales per measurement will be evaluated based on the method implemented in the software program (SAS version 9.2 or higher, SAS Institute Inc) provided along with the questionnaire. Change from baseline will be evaluated for all summary scales after 6 and 12 months as the primary endpoint. BDI, effective contact position (with respect to DRT and AC-PC coordinates) and the volume of activated tissue will be analyzed similar to the primary endpoint using linear regression.

Rates of recruitment per month, screening failures, and drop-out from the trial will be evaluated and considered for the confirmative trial. All secondary analyses will be exploratory.

Safety analyses will be performed for all patients for whom 1 of the treatments was started. Patients will be analyzed according to treatment received. Rates of adverse events and serious adverse events will be calculated with corresponding 2-sided 95% confidence intervals.

### Ethical Issues

An adequate subject insurance contract has been taken out. The study protocol has been approved by the independent ethics committee of the University of Freiburg (EK 207/15). The study will be conducted in accordance with the ethical principles of the Declaration of Helsinki and applicable regulatory requirements. The DISTINCT trial has been registered in the German Clinical Trials Register (DRKS00008913) and at ClinicalTrials.gov (NCT02491554).

## Results

Recruitment to the DISTINCT trial opened in September 2015 and is expected to close in June 2017. At the time of manuscript submission the trial is open to recruitment.

## Discussion

The DISTINCT trial is an investigator-initiated, randomized, controlled, observer-blinded trial comparing DTI tractography-assisted stereotactic surgery with a target point of the DRT in general anesthesia to stereotactic surgery of the thalamic/subthalamic region as conventionally used. It is hypothesized that DTI tractography-assisted stereotactic surgery in general anesthesia is safe and is equal to conventional AC-PC coordinate-based stereotactic surgery with intraoperative testing in the awake patient.

To examine the 2 discrete treatment groups, a randomized controlled parallel group study is the appropriate design. Efforts to blind patients would annihilate favorable effects in the tractography-based treatment group (single trajectory, shorter duration of surgery), so a rater-blinded design is implemented. Reliability is enhanced by double rating of video-documented tremor assessments by 2 independent raters.

Chen et al [15] retrospectively analyzed a series of AC-PC coordinate-based Vim-DBS implantations in general anesthesia and compared it to implantations in awake patients. As clinical outcome parameter, a self-evaluation with a mailed questionnaire was used which led to a variable follow-up latency and a considerable drop-out rate. As a contrast, the focus of our study is the DTI tractography-assisted Vim-DBS implantation. Furthermore, by means of a randomized controlled trial and clinical tremor assessment more valid data can be yielded.

DTI tractography of the DRT can generally be achieved with probabilistic [21] or deterministic [10,11] fiber tracking; however, only the latter is available for stereotactic planning in CE-approved planning systems. As a result, this study employs deterministic fiber tracking.

Tractography with a seed ROI in the ipsilateral dentate nucleus and target ROI in the ipsilateral precentral gyrus reliably yields the nondecussating fibers of the DRT described by Meola et al [22]. However, as the decussating fibers cross below the level of the red nucleus and converge with the nondecussating fibers before entering the thalamus, the issue of decussation has little relevance to the stimulation target site in the subthalamic region.

Although it is generally agreed on that effects of DBS are exerted on fibers, there is only limited published evidence on DTI tractography-assisted stereotactic surgery [23]. A post hoc study of our group in 11 patients who received Vim-DBS was able to show that effective electrode positions are located inside or within proximity of the DRT [12], extending knowledge gathered by Klein et al [24] and Coenen et al [10,11]. The DISTINCT study is the first to take advantage of this recently tractographically identified target structure for treatment of medically refractory tremor. As a single-center pilot feasibility trial, the results can be limited in validity; however, it has the potential to serve as a cornerstone for the evolving technique of DTI tractography-assisted stereotactic surgery.

## Abbreviations

AC-PC: anterior commissure–posterior commissure
BDI: Beck Depression Inventory
CT: computed tomography
DBS: deep brain stimulation
DISTINCT: Deep Brain Stimulation for Tremor Tractographic Versus Traditional
DRT: dentatorubrothalamic tract
DTI: diffusion tensor imaging
EPI: echo-planar imaging
FTMTRS: Fahn-Tolosa-Marin Tremor Rating Scale
ITT: intention to treat
MDRS: Mattis Dementia Rating Scale
MRI: magnetic resonance imaging
QUEST: Quality of Life in Essential Tremor Questionnaire
ROI: region of interest
SE: spin-echo
SF-36: Short Form 36
TE: echo time
TR: repetition time
Vim: thalamic ventral intermediate nucleus

## References

[1] Zappia M, Albanese A, Bruno E, Colosimo C, Filippini G, Martinelli P, Nicoletti A, Quattrocchi G, Abbruzzese G, Berardelli A, Allegra R, Aniello MS, Elia AE, Martino D, Murgia D, Picillo M, Squintani G (2013). Treatment of essential tremor: a systematic review of evidence and recommendations from the Italian Movement Disorders Association. J Neurol.

[2] Koller W, Biary N, Cone S (1986). Disability in essential tremor: effect of treatment. Neurology.

[3] Chopra A, Klassen BT, Stead M (2013). Current clinical application of deep-brain stimulation for essential tremor. Neuropsychiatr Dis Treat.

[4] Benabid AL, Pollak P, Gao D, Hoffmann D, Limousin P, Gay E, Payen I, Benazzouz A (1996). Chronic electrical stimulation of the ventralis intermedius nucleus of the thalamus as a treatment of movement disorders. J Neurosurg.

[5] Hassler R, Mundinger F, Riechert T (1979). Stereotaxis in Parkinson Syndrome.

[6] Spiegelmann R, Nissim O, Daniels D, Ocherashvilli A, Mardor Y (2006). Stereotactic targeting of the ventrointermediate nucleus of the thalamus by direct visualization with high-field MRI. Stereotact Funct Neurosurg.

[7] Vassal F, Coste J, Derost P, Mendes V, Gabrillargues J, Nuti C, Durif F, Lemaire J (2012). Direct stereotactic targeting of the ventrointermediate nucleus of the thalamus based on anatomic 1.5-T MRI mapping with a white matter attenuated inversion recovery (WAIR) sequence. Brain Stimul.

[8] Henderson JM (2012). “Connectomic surgery”: diffusion tensor imaging (DTI) tractography as a targeting modality for surgical modulation of neural networks. Front Integr Neurosci.

[9] Hamel W, Herzog J, Kopper F, Pinsker M, Weinert D, Müller D, Krack P, Deuschl G, Mehdorn HM (2007). Deep brain stimulation in the subthalamic area is more effective than nucleus ventralis intermedius stimulation for bilateral intention tremor. Acta Neurochir (Wien).

[10] Coenen VA, Allert N, Mädler B (2011). A role of diffusion tensor imaging fiber tracking in deep brain stimulation surgery: DBS of the dentato-rubro-thalamic tract (DRT) for the treatment of therapy-refractory tremor. Acta Neurochir (Wien).

[11] Coenen VA, Mädler B, Schiffbauer H, Urbach H, Allert N (2011). Individual fiber anatomy of the subthalamic region revealed with diffusion tensor imaging: a concept to identify the deep brain stimulation target for tremor suppression. Neurosurgery.

[12] Coenen VA, Allert N, Paus S, Kronenbürger M, Urbach H, Mädler B (2014). Modulation of the cerebello-thalamo-cortical network in thalamic deep brain stimulation for tremor: a diffusion tensor imaging study. Neurosurgery.

[13] Fenoy AJ, Simpson RK (2012). Management of device-related wound complications in deep brain stimulation surgery. J Neurosurg.

[14] Zrinzo L, Foltynie T, Limousin P, Hariz MI (2012). Reducing hemorrhagic complications in functional neurosurgery: a large case series and systematic literature review. J Neurosurg.

[15] Chen T, Mirzadeh Z, Chapple K, Lambert M, Dhall R, Ponce FA (2016). “Asleep” deep brain stimulation for essential tremor. J Neurosurg.

[16] Fahn S, Tolosa E, Marin C, Jankovic J, Tolosa E (1993). Clinical Rating Scale for Tremor. Parkinson's Disease and Movement Disorders. Second edition.

[17] Deuschl G, Bain P, Brin M (1998). Consensus statement of the Movement Disorder Society on tremor. Mov Disord.

[18] Tröster AI, Pahwa R, Fields JA, Tanner CM, Lyons KE (2005). Quality of life in essential tremor questionnaire (QUEST): development and initial validation. Parkinsonism Relat Disord.

[19] Zaitsev M, Hennig J, Speck O (2004). Point spread function mapping with parallel imaging techniques and high acceleration factors: fast, robust, and flexible method for echo-planar imaging distortion correction. Magn Reson Med.

[20] Julious SA (2005). Sample size of 12 per group rule of thumb for a pilot study. Pharmaceut Statist.

[21] Kwon HG, Hong JH, Hong CP, Lee DH, Ahn SH, Jang SH (2011). Dentatorubrothalamic tract in human brain: diffusion tensor tractography study. Neuroradiology.

[22] Meola A, Comert A, Yeh F, Sivakanthan S, Fernandez-Miranda JC (2016). The nondecussating pathway of the dentatorubrothalamic tract in humans: human connectome-based tractographic study and microdissection validation. J Neurosurg.

[23] Torres CV, Manzanares R, Sola RG (2014). Integrating diffusion tensor imaging-based tractography into deep brain stimulation surgery: a review of the literature. Stereotact Funct Neurosurg.

[24] Klein JC, Barbe MT, Seifried C, Baudrexel S, Runge M, Maarouf M, Gasser T, Hattingen E, Liebig T, Deichmann R, Timmermann L, Weise L, Hilker R (2012). The tremor network targeted by successful VIM deep brain stimulation in humans. Neurology.

